# Factors Influencing the Measurement of Plasma/Serum Surfactant Protein D Levels by ELISA

**DOI:** 10.1371/journal.pone.0111466

**Published:** 2014-11-03

**Authors:** Preston E. Bratcher, Amit Gaggar

**Affiliations:** 1 Department of Medicine and Division of Pulmonary, Allergy, and Critical Care Medicine, University of Alabama at Birmingham, Birmingham, Alabama, United States of America; 2 Gregory Fleming James Cystic Fibrosis Research Center, University of Alabama at Birmingham, Birmingham, Alabama, United States of America; 3 University of Alabama at Birmingham, UAB Lung Health Center, Birmingham, Alabama, United States of America; 4 Medicine Service, United States Department of Veterans Affairs Medical Center, Birmingham, Alabama, United States of America; 5 Program in Protease and Matrix Biology, University of Alabama at Birmingham, Birmingham, Alabama, United States of America; The Hospital for Sick Children and The University of Toronto, Canada

## Abstract

**Background:**

Extensive variations in human surfactant protein D (SP-D) levels in circulation as measured by ELISA exist in the published literature. In order to determine the source of these variations, factors influencing the measurement by ELISA were explored.

**Materials and Methods:**

Peripheral blood from healthy individuals was collected into various vacutainers during the same blood draw. Recombinant SP-D was diluted into different matrices and used for a standard curve. Samples were analyzed by capture ELISA using one of two distinct detection antibodies.

**Results:**

The type of matrix had some effects on detection of recombinant SP-D. The type of anticoagulant used and dilution factor had very little effect, except for in plasma collected in EDTA vacutainers. The extent of variation in published values seemed to be due to the ELISA configuration employed, and, in agreement with this, we found that by switching the detection antibody, there was a 50% decrease in the extrapolated SP-D value of serum and plasma samples. Storage of samples resulted in slight changes in measured SP-D levels.

**Conclusions:**

The ELISA configuration employed to measure circulating levels of SP-D has a significant effect on the extrapolated values. In both configurations tested, the use of EDTA as a coagulant resulted in inconsistent values, and we, therefore, suggest the avoidance of this anticoagulant when assaying for SP-D by ELISA. While the demonstrated effects of several factors on measurement of SP-D may not account for all the disparities amongst the previous studies, they stress that variations in methodologies for measuring the same protein can result in very inconsistent results.

## Introduction

Surfactant protein D (SP-D) is a pulmonary collectin involved in regulation of inflammation, innate immune defense, and surfactant homeostasis. It is expressed by Clara cells and alveolar type II cells in the lung. SP-D has a multimeric structure which gives it the ability to agglutinate pathogens, as well as aid in the clearance of apoptotic cells, cellular debris, and foreign particles in the lung [reviewed in [1]].

Circulating levels of SP-D have been examined for their potential use as a biomarker in various diseases including dermatitis [2], [3], acute lung injury (ALI)/acute respiratory distress syndrome (ARDS) [4]–[13], periodontitis [14], interstitial pulmonary fibrosis (IPF) [10], [12], [15]–[23], chronic obstructive pulmonary disease (COPD) [15], [24]–[36], emphysema [37], cystic fibrosis (CF) [15], [38], [39], coronary disease [40], [41], sclerosis [42]–[46], cancer [47], [48], sarcoidosis [21], [49], allergies [28], [50]–[52], rheumatoid arthritis [53], [54], and respiratory infections [18], [55]–[60]. SP-D levels have also been proposed to correlate with genetic elements [61]–[63], body mass index (BMI) [64]–[68], age [65], circadian rhythm [69], and with particle exposure [70],[71] and cigarette smoking habits [25], [27], [28], [31], [37], [72]–[77]. In addition, there have been studies examining the levels of SP-D in subjects with Turner syndrome [78], paraquat intoxication [79], swimming in variably treated waters [80], lung transplant patients [81], patients undergoing neurosurgical operations [82], drowning victims [83], polymyositis/dermatomyositis [84], dementia [85], lupus [86], and sleep apnea [87].

Similarly to CC-16 and KL-6, SP-D is thought to be a marker of pulmonary leak into the vasculature [88], and therefore alveolar destruction would result in an increase in levels of these pulmonary proteins in the blood. However, protein levels in lung do not always correlate with protein levels in blood [35], suggesting the possibility of alternative mechanisms affecting SP-D levels in circulation.

Various commercially available kits and non-commercially available ELISA configurations have been used to compare SP-D levels in plasma and serum from normal, healthy controls and the various disease states described above. Interestingly, there is a very substantial discrepancy between the reported values of the healthy control populations between studies, as well as in the magnitude of the range of values in this population. While the ELISA configuration used to measure SP-D seemed to have a large impact on the values reported, there are significant variations in the healthy control SP-D levels and range amongst reports using the same configuration. The purpose of this study is to determine factors affecting the measurement of SP-D by ELISA that may, therefore, explain the variations of serum and plasma SP-D levels reported in the published literature.

## Materials and Methods

### Study subjects, peripheral blood collection, and processing

**Figure 2:**
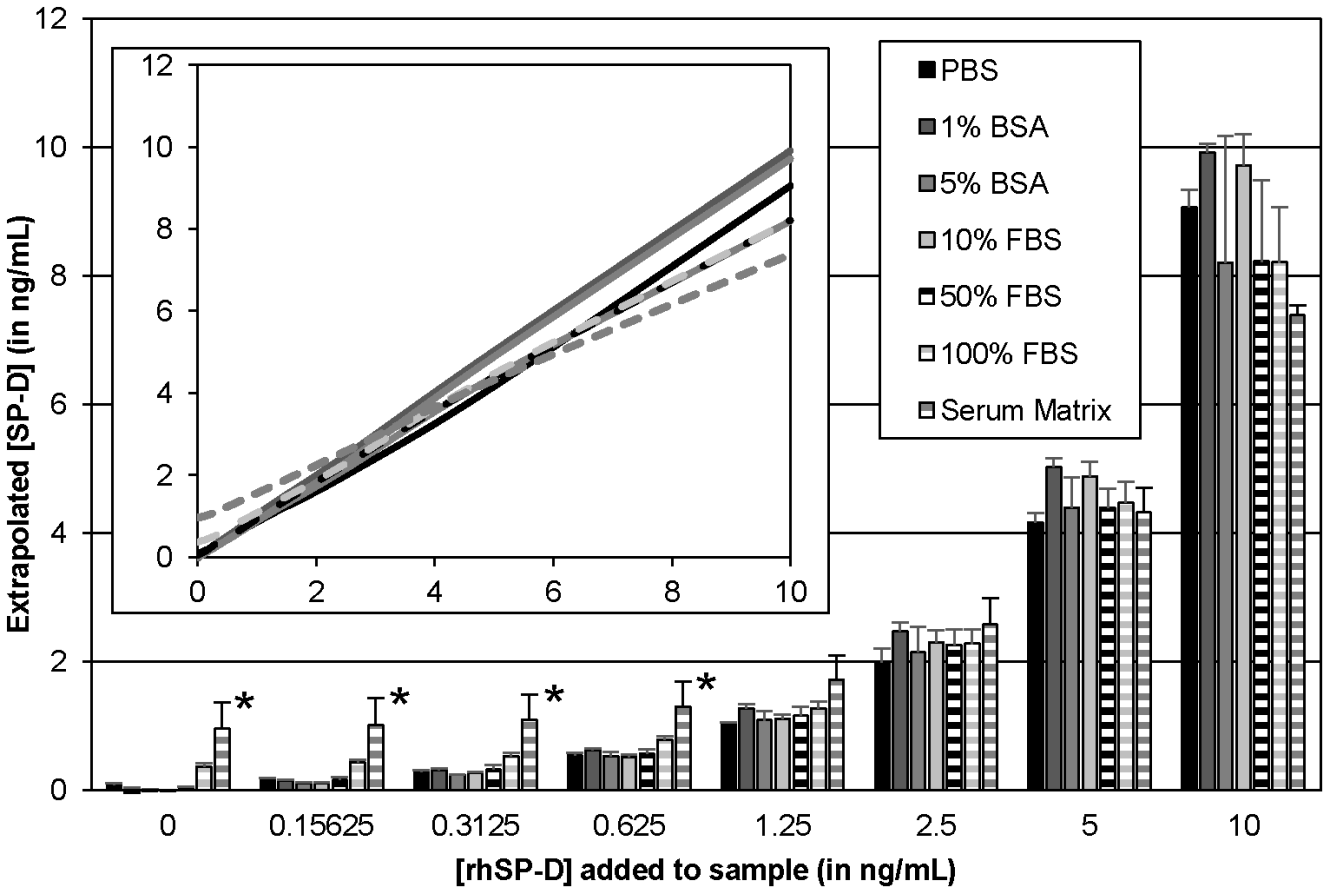
Use of various diluents for the recombinant SP-D standard. A 1 µg/mL recombinant SP-D stock was diluted 1∶99 in various matrices and then serially diluted 2 fold in the same matrix. Mean and standard deviation for three independent experiments are shown. An asterisk (*) denotes values are significantly different (p≤0.01) from both the 1% BSA and 10% FBS values by one-way ANOVA using Tukey's multiple comparison test.

**Figure 4:**
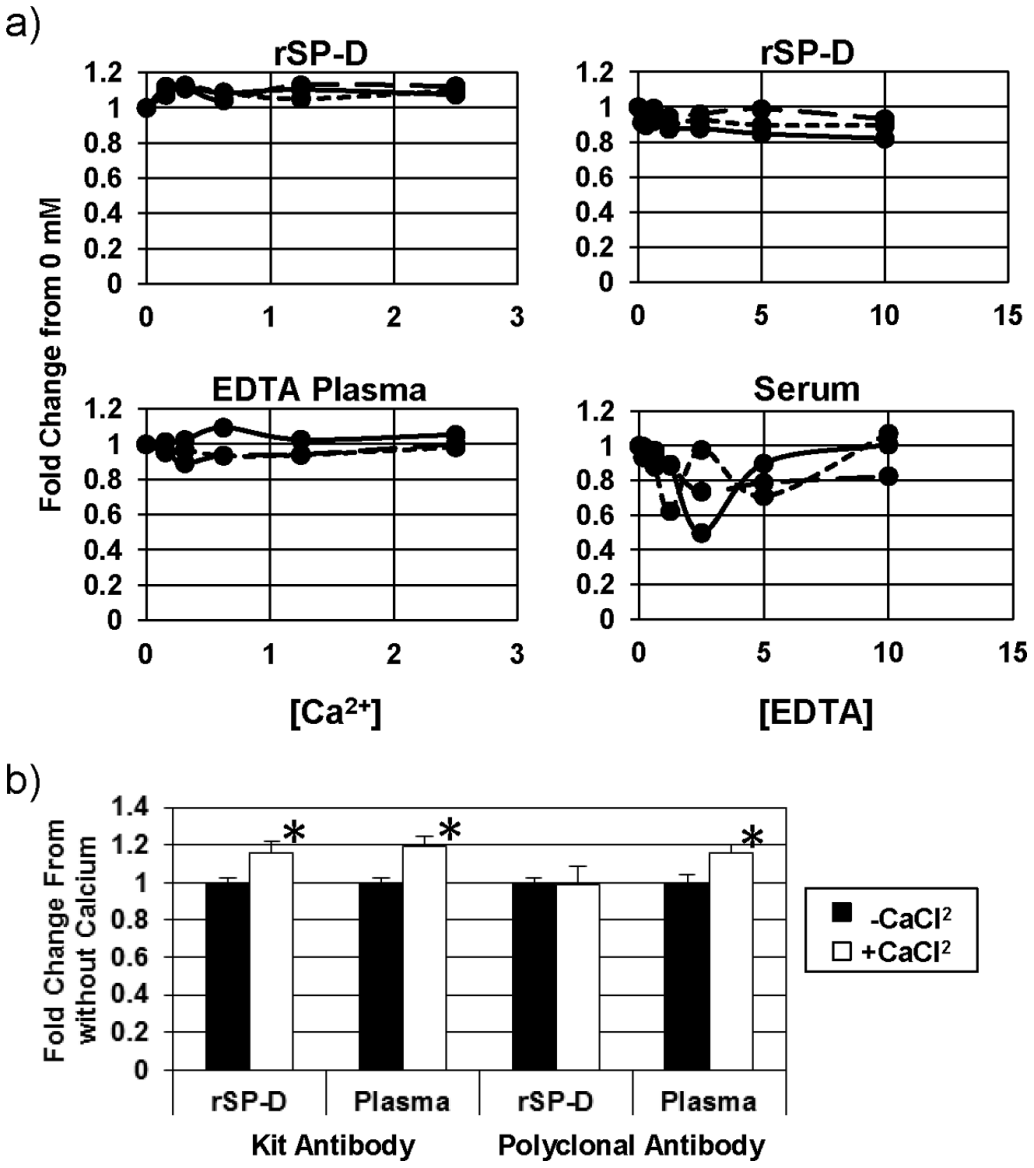
Influence of calcium concentration on detection of recombinant SP-D and native SP-D in serum and plasma. a) Recombinant SP-D (rSP-D), serum, and EDTA plasma were assayed in twofold dilutions of [Ca^2+^] or [EDTA] (concentrations are displayed in mM). For the inclusion of calcium with recombinant SP-D, Hank's Balanced Salt Solution (HBSS) was used as a buffer instead of PBS. Recombinant SP-D was assayed at 5 ng/mL while serum and plasma were diluted twofold. Each line represents an independent experiment. b) ELISAs were performed with detection reagents diluted in HBSS without calcium chloride (-CaCl^2^) or with 5 mM calcium chloride (+CaCl^2^). Wash buffer also either lacked or included calcium chloride. Recombinant SP-D was assayed at 10 ng/mL and Heparin plasma from four subjects was pooled, and sample values were extrapolated from a single standard curve under the conditions lacking calcium. n = 3 samples per group.

All human studies were approved by the University of Alabama at Birmingham Institutional Review Board for Human Use with all subjects providing written consent. Peripheral blood was collected from healthy volunteers by venipuncture into serum, heparin sulfate, K_2_EDTA, and sodium citrate vacutainers (BD Biosciences) during a single draw. Samples were kept at room temperature until blood in the serum tube was coagulated. Afterwards, samples were centrifuged at 400xg for 10 minutes to separate blood cells from serum or plasma. Samples were either directly aliquoted and stored at −80°C or given an additional round of centrifugation at 3000xg for 10 minutes to separate platelets from serum or plasma. All serum and plasma sample values depicted in figures were free of platelets. All experiments had 6 samples per group except for the experiments depicted in Figures 2 and 4.

### Measurement of SP-D concentration by ELISA and data analysis

Mouse antihuman SP-D capture antibody, biotinylated mouse antihuman SP-D detection antibody, streptavidin conjugated to horse radish peroxidase, and recombinant human SP-D standard were purchased as a kit from R&D Systems (Catalog # DY1920) and used according to the manufacturer's protocol. For experiments testing the effects of different matrices on the detection of recombinant human SP-D, a concentrated stock was made in PBS with 1% BSA from separately purchased recombinant human SP-D expressed in NSO cells (R&D Systems, Catalog # 1920-SP-050). For studies comparing detection antibodies, polyclonal goat antihuman SP-D antibody (R&D systems, Catalog # AF1920) was used instead with a monoclonal mouse antigoat conjugated to horse radish peroxidase (Sigma) used as a secondary. All sample values were extrapolated from a second order polynomial curve fit of 9 concentrations of the standard (two-fold dilutions with a high concentration of 40 ng/mL) diluted in 1% BSA PBS. Statistical analyses were performed as described in the figure legends using Prism 5 (GraphPad Software), and all reported p values are two-tailed. All error bars represent standard deviation.

## Results

### Variations in previously reported SP-D levels

**Figure 1:**
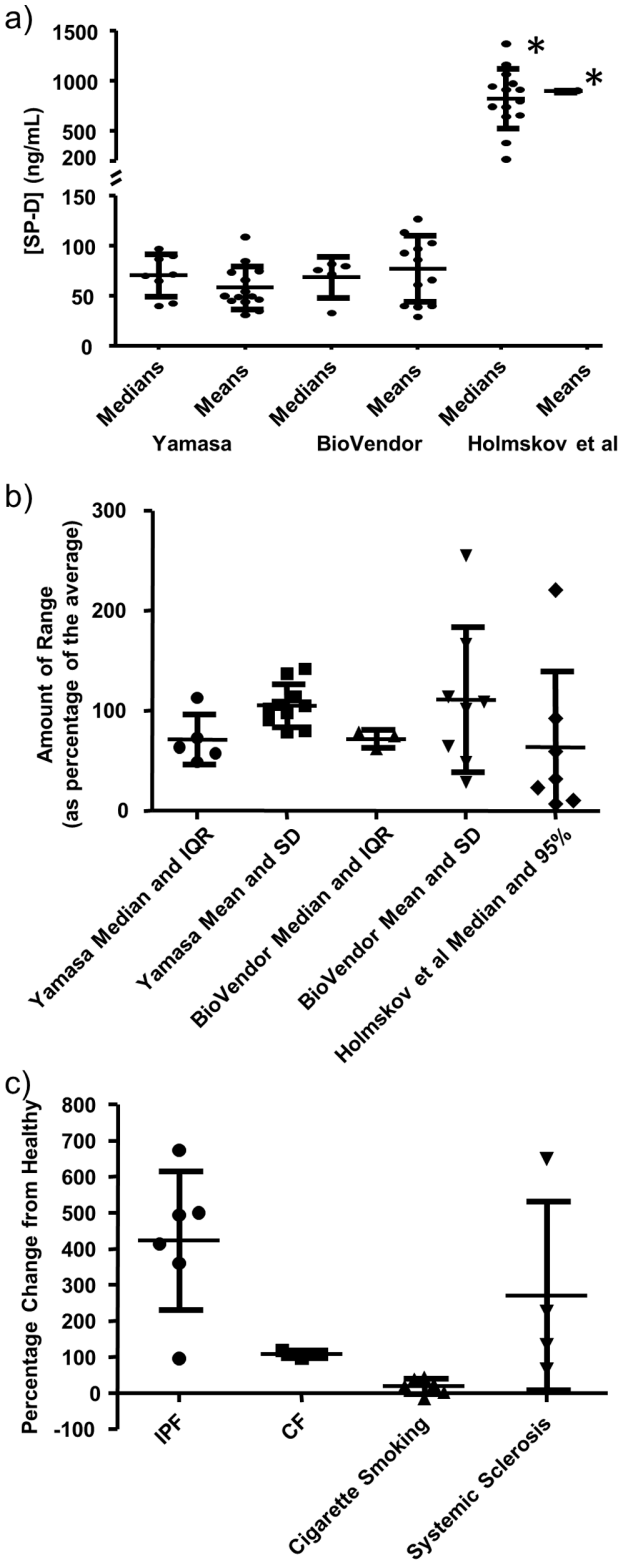
Published values for [SP-D] in the blood. a) A substantial amount of variation in the average [SP-D] in the serum/plasma of healthy control population exists between studies using the same or different ELISA configurations. Three configurations (Yamasa [12], [13], [17]–[19], [21], [25], [28], [38], [43]–[45], [49], [50], [59], [61], [77], [84], [89], BioVendor [14], [31]–[36], [46], [52], [56], [64], [65], [72], [75], [76], [79], [80], and Holmskov et al. [3], [39], [53]–[55], [58], [62], [66], [69], [70], [78], [85], [86]) were compared. b) The range of healthy control [SP-D] greatly varied from study to study. Values shown are either median and interquartile range (IQR), median and 95% confidence interval (95%), or mean and standard deviation (SD). c) The calculated fold increase from the average healthy [SP-D] and average [SP-D] during IPF [12], [16]–[19], [21], CF [15], [38], [39], cigarette smoking [28], [31], [72]–[75], or sclerosis [42]–[44], [46] was different between publications. An asterisk (*) denotes p≤0.001 by one-way ANOVA with Tukey's multiple comparison test.

In order to determine the mean and range of SP-D levels in the serum/plasma of normal, healthy individuals, we performed a non-exhaustive search of the literature which revealed more than 60 publications in which these values were described. Interestingly, an unexpected amount of variation on these reported values was observed. When grouping according to the ELISA configuration used, one configuration consistently resulted in significantly higher values than the two other configurations for which multiple references were obtained (Figure 1a). For each configuration, a substantial range of means/medians was observed in the healthy control population. In addition to the variations seen in the averages, the spectrum of values in all populations ranged from 55.9 pg/mL [22] to 3.9653 µg/mL [89], representing a more than 70,000 fold difference. When examining the range of values seen in healthy subjects from each individual study and grouping according to the ELISA configuration used, a large variation in range was observed (Figure 1b). Overall, the study with the smallest range for any population (as a percentage of the average) reported a 95% confidence interval of 95.7–109.0 [54], while the study containing the population with the largest range reported a standard deviation of 327% of the mean value [42].

Based on the above observations, we can infer that the largest difference in the measured levels of SP-D is due to the ELISA configuration employed, but that there are still significant differences between the averages of healthy individuals from studies using the same configurations. In order to compare the results of various studies in another manner, we examined the fold change from healthy populations for the four diseases/conditions for which a substantial number of publications were available (IPF, CF, cigarette smoking, and systemic sclerosis). This would allow us to control for ELISA configurations employed, technical protocol followed for the ELISA as well as for sample collection and processing, and any differences in the populations (as controls have been appropriately matched). The expectation was that the fold change from healthy would be very similar for each disease/condition. However, large differences in the extent of this change were observed (Figure 1c).

### Influence of matrix on detection of recombinant SP-D

In order to determine if the matrix used to generate the serial dilutions of recombinant human standard (rhSP-D) had any effects upon measurement, we compared values produced by dilutions in PBS, solutions of BSA (in PBS), and solutions of FBS (diluted in PBS) (Figure 2). At higher concentrations of protein (i.e. 5% BSA, 50% FBS, and 100% FBS), a decrease in the amount of rhSP-D detected was observed, with the amount measured in samples containing 10 ng/mL rhSP-D being the most variable of any samples measured in this assay. Using the manufacturer's recommended matrix (1% BSA) produced results very similar to a 10% FBS matrix, with both giving very consistent measurements and the greatest values. The values for PBS matrix gave similarly consistent measurements, but at a slightly lower value. This effect may be due to adsorption of rhSP-D without a carrier protein to the tubes used for serial dilutions [90]. Serum Matrix (Millipore), which had background levels of SP-D, also inhibited detection of recombinant SP-D at higher concentrations. All further standard curves were established by serial dilutions of recombinant SP-D in 1% BSA in PBS.

### Influence of anticoagulant on extrapolated SP-D level

**Figure 3:**
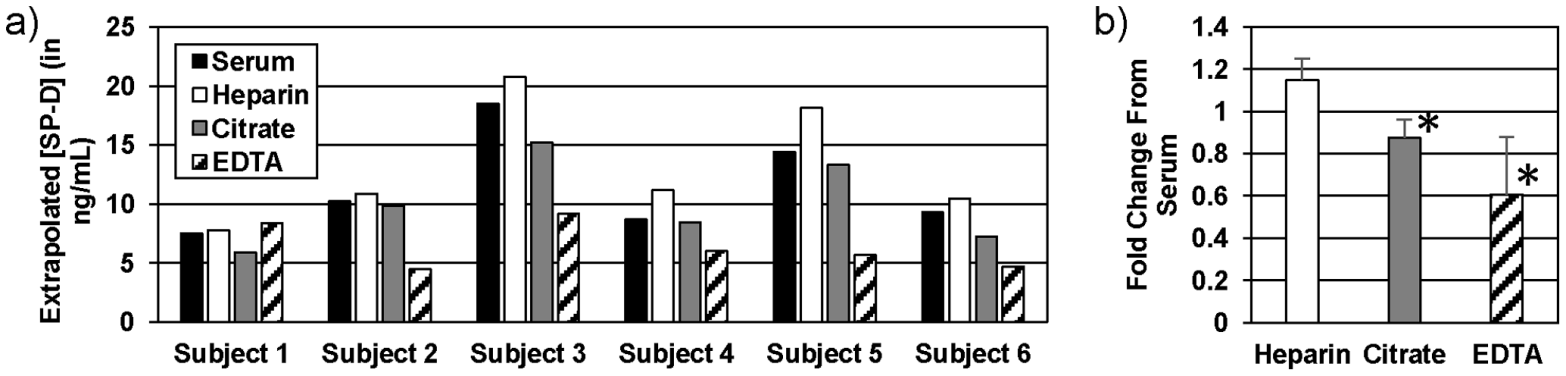
Detection of SP-D in serum and plasma collected using various anticoagulants. a) SP-D concentrations were measured in samples collected into four different vacutainers during a single blood draw. b) Measured values of samples were normalized to the SP-D concentration in serum for each patient in order to compare the effect of the anticoagulant on the measured SP-D concentration. An asterisk (*) denotes values are significantly different by Wilcoxon signed rank test (for EDTA, p = 0.0156, and for Citrate, p = 0.0078).

One factor that was found to differ between studies and could, therefore, be a source of variation of reported healthy population values, was the type of anticoagulant (or lack thereof) in the collection container. When blood was simultaneously collected in various vacutainers, serum and heparin plasma gave similar measurements of SP-D, while citrate plasma gave values significantly lower than serum values (Figure 3). EDTA plasma gave the most inconsistent results, and values were also significantly lower than serum values.

### Influence of calcium on detection of SP-D by ELISA

In order to determine if calcium concentration in the sample had a significant effect upon the measurement of SP-D, rhSP-D was assayed in serially diluted CaCl_2_ or EDTA. The detection of rhSP-D was only slightly effected by the changes in calcium concentration in the sample (Figure 4a). Additionally, we examined the effects of calcium concentration in the human samples by adding calcium to EDTA plasma samples and by adding EDTA to serum samples. While reconstituting the free calcium in the EDTA samples had little effect, the addition of EDTA to the serum samples had a dramatic effect that was inconsistent amongst the patient samples.

While many ELISAs employ the use of PBS lacking divalent cations as a buffer during the detection of antibody-captured antigens, the ELISA configuration employed by Holmskov et al. uses a Tris-buffered saline solution containing 5 mM calcium chloride, as the monoclonal detection antibody binds to SP-D in the presence of calcium [58]. In addition, it was previously demonstrated that SP-D has the ability to bind to various immunoglobulins in a calcium-dependent manner [91]. In order to determine the effects of calcium in the context of SP-D detection by ELISA, ELISAs were performed with antibodies diluted in buffer with or without calcium. We examined these effects on both recombinant SP-D and SP-D in plasma with either the ELISA kit's detection antibody or a polyclonal goat anti-SP-D antibody produced by the same manufacturer. In all cases but one, the addition of 5 mM calcium to the dilution and wash buffer resulted in a small (∼17%) but significant increase in SP-D concentration relative to detection in the absence of calcium (Figure 4b). It is important to note that with the kit reagents, since the relative increase in detection of recombinant SP-D and SP-D in plasma in the presence of calcium is very similar, the extrapolated SP-D concentration in plasma when using a standard curve of recombinant SP-D should not significantly change. Interestingly, while the inclusion of calcium increased the recognition of native SP-D by the polyclonal antibody, it had no effect on the detection of recombinant SP-D by this antibody. Although it is beyond the scope of this study, future work will explore whether this effect is due to an increase in antibody recognition of antigen, increase in non-specific binding of antibodies by captured SP-D, or an increase through another mechanism.

### Influence of detection antibody on extrapolated SP-D level

**Figure 5:**
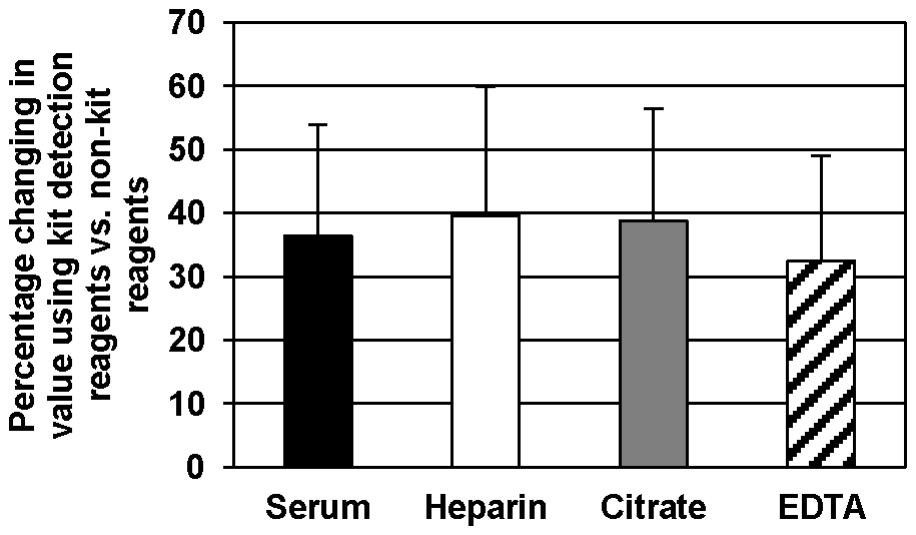
Comparison of SP-D values using two different detection antibodies. Serum, plasmas, and recombinant SP-D standard were detected using either kit reagents or non-kit reagents as described in the methods. SP-D concentrations in human samples were extrapolated from the standard curve as detected with the corresponding reagents. All values were significantly different (p<0.05) by Wilcoxon signed rank test.

Given that some of the variation seen in the published literature might be explained by the use of different antibodies, we detected SP-D in serum and plasma samples using either the ELISA kit's detection antibody or the polyclonal goat anti-SP-D antibody. While there is no significant difference between detection by the kit antibody versus the polyclonal goat antibody with regard to the type of collection tube used, overall, there is a ∼50% reduction in the extrapolated value produced by detection with the polyclonal goat antibody compared to the kit antibody (Figure 5). In both cases, the same capture antibody was employed, and it is therefore possible that using a different capture antibody could have a similar effect on varying the extrapolated SP-D concentration in the sample.

### Influence of sample dilution on extrapolated SP-D level

**Figure 6:**
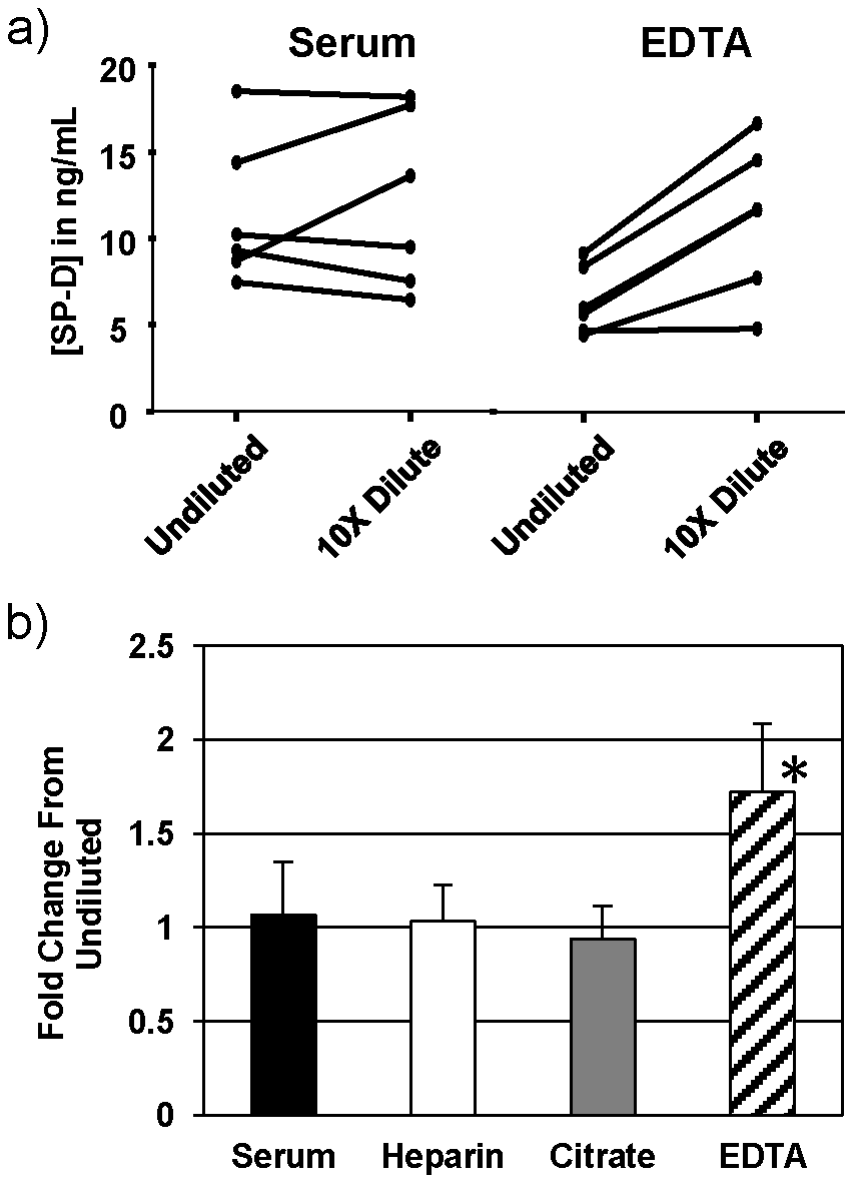
Comparison of SP-D values using neat and diluted samples. a) Serum and plasma samples were assayed as both undiluted and diluted tenfold in PBS. The extrapolated value in the tenfold diluted sample was multiplied by 10 in order to compare to the original, undiluted sample. b) Measured values of diluted samples were normalized to the SP-D concentration in the corresponding undiluted sample in order to compare the effect of the anticoagulant on the measured SP-D concentrations in these conditions. An asterisk (*) denotes p = 0.0313 by Wilcoxon signed rank test.

Another factor that fluctuated from study to study was whether the sample was assayed neat or the sample was prediluted (and the amount the sample was diluted). Given this fact and that diluting recombinant SP-D into 100% FBS had some influence in the detection of said SP-D, we compared SP-D values detected in undiluted and diluted samples. There was a very minimal difference between values produced by undiluted and 10 fold diluted serum, citrate plasma, and heparin plasma (Figure 6). Ten fold diluted EDTA plasma, however, produced a significantly higher extrapolated SP-D value when compared to the same sample undiluted. This same effect was seen when the polyclonal goat antibody tested above was used for detection (data not shown).

### Influence of storage condition on extrapolated SP-D level

**Figure 7:**
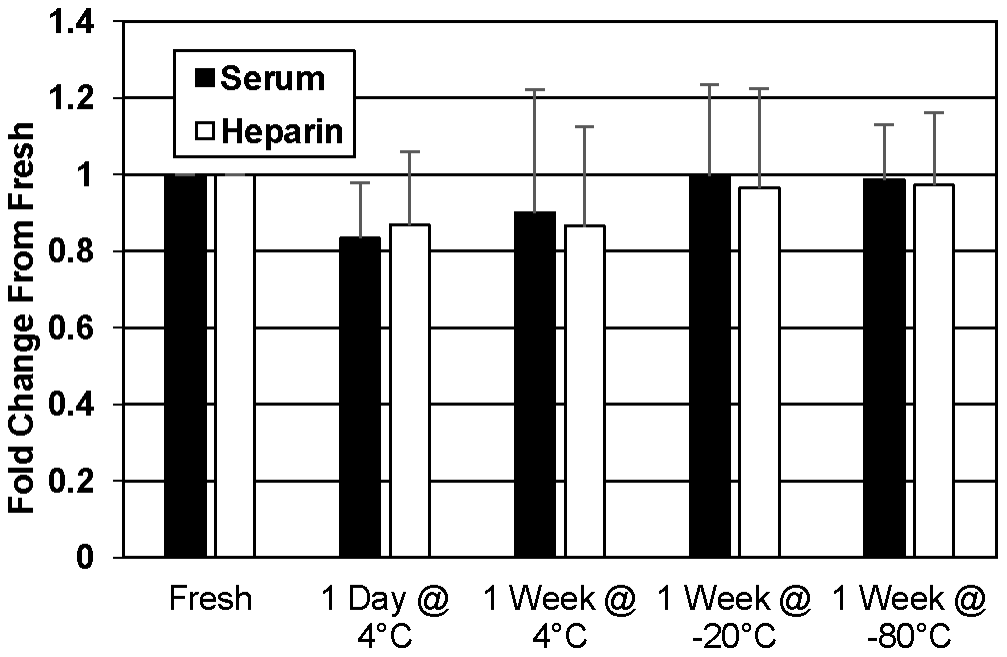
Comparison of SP-D values in samples stored in various conditions. Serum and heparin plasma samples collected during a single draw were either analyzed fresh or aliquoted and stored at 4°C, −20°C, or −80°C.

While the most common processing technique involved separating the blood cells (but not platelets) from serum and plasma and storing this sample at −80°C until assayed, some variations in processing and storage were present. To address this, we assayed serum and heparin plasma without platelets immediately after processing or after storage at 4°C, −20°C, or −80°C (Figure 7); there were no significant differences between conditions. Additionally, there were only subtle differences between a sample stored at −20°C and −80°C, and depending on whether the sample went through a freeze/thaw (FT) cycle, contained platelets or not, or was spun after thawing to separate any precipitates/debris (data not shown). SP-D concentrations of samples stored at −20°C and −80°C for two weeks were not different from the same samples at one week (data not shown).

## Discussion

This study provides experimental evidence that variations in the anticoagulant used and ELISA configuration can have dramatic effects upon the measured SP-D level. Storage and processing of samples as well as diluent used for the standard have minor effects on the level of SP-D extrapolated by ELISA. Although these results may partially explain the variations seen in reported SP-D values in the blood, it is important to note the caveats associated with this review of the literature. For the comparison of healthy control groups amongst various studies, it is possible that differences in ethnic background and/or geographical location from which subjects were recruited may contribute to the variations seen when the same ELISA configuration was employed, as could the type of anticoagulant. However, we would expect the range of samples between healthy populations of different studies to be similar. In addition, assuming that an appropriately matched control population was used for comparison to the disease population, the fold change between healthy and disease should be more similar than what was observed in the literature.

Measuring SP-D by ELISA presents a problem due to its multimeric structure. For each molecule (a complete dodecamer) of multimerized protein, there are at least 12 of the same epitopes present. Due to this property, it is not necessary to use discrete antibodies in a sandwich ELISA format to capture and then detect SP-D; even the same monoclonal antibody could be used for both. However, this may cause a difference in read out compared to using antibodies that detect different epitopes, due to the fact that the degree of multimerization has been shown to vary in the lungs during different disease states [92] and using the same monoclonal antibody would fail to detect a monomer. Even using two monoclonals that recognize distinct epitopes to measure SP-D in its native state would be confounded by differences in the degree of multimerization. For example, if an antibody against the carbohydrate recognition domain of SP-D was used to capture and an antibody against the neck region of SP-D was used to detect, a single, captured monomer of SP-D would be able to bind a single anti-neck antibody. However, a single, captured dodecamer would be able to bind 12 anti-neck antibodies. In order to accurately establish absolute concentrations by ELISA, it would be important that the multimeric structure of the standard was equivalent to that of the samples, as there could be differences in antigen binding in higher order multimers due to steric hinderance. Unfortunately, we did not have the necessary resources to examine the influence of the degree of multimerization on ELISA measurements.

In addition to the possible effects caused by its multimeric nature, there is evidence in the literature to variable post-translational modifications of SP-D in native samples. These modifications may, in fact, mask the epitope recognized by an ELISA antibody. This may account for one of the largest differences seen in the reported ELISA measurements of SP-D in serum/plasma, as the highest concentrations of SP-D came from the non-commercially available ELISA which used purified, native SP-D as a standard; this is opposed to almost all others, which used recombinantly expressed SP-D as a standard. Regardless of whether the antibodies used were raised against recombinant SP-D or purified native, because the post-translational modifications can vary from one patient to the next, this presents a significant obstacle to accurate measurements of natural SP-D using antibodies.

The results of this study demonstrate the influence that the antibody may have on the SP-D concentration as measured by ELISA. In our experiments, it is important to note that the same recombinant standard and human serum/plasma samples were analyzed. Given the confounders mentioned above, it is possible that the antibody from the kit recognizes an epitope that is not post-translationally modified or altered in human serum/plasma SP-D, while several of the epitopes recognized by the polyclonal antibody are modified in human serum/plasma SP-D, but were unmodified in the recombinantly expressed SP-D (which was used as the immunogen). This would result in the observed relative decrease in binding of the native SP-D from the blood compared to the recombinant SP-D.

Accuracy of SP-D measurements is critically important to the validation of this protein as a biomarker in pulmonary disease. Standardization of sample processing and storage, including the avoidance of EDTA as an anticoagulant, is necessary to ensure consistent results. Although absolute values may vary greatly due to the ELISA configuration employed, relative differences in SP-D concentrations amongst various disease groups should be consistent throughout the published literature. We are currently exploring alternative methods to quantify SP-D levels in the blood, as we believe this is necessary in order to establish the absolute concentration.
